# *Acremonium terricola* Culture’s Dose–Response Effects on Lactational Performance, Antioxidant Capacity, and Ruminal Characteristics in Holstein Dairy Cows

**DOI:** 10.3390/antiox11010175

**Published:** 2022-01-17

**Authors:** Fanlin Kong, Yijia Zhang, Shuo Wang, Zan Cao, Yanfang Liu, Zixiao Zhang, Wei Wang, Na Lu, Shengli Li

**Affiliations:** 1 Beijing Engineering Technology Research Center of Raw Milk Quality and Safety Control, The State Key Laboratory of Animal Nutrition, Department of Animal Nutrition and Feed Science, College of Animal Science and Technology, China Agricultural University, No. 2 Yuanmingyuan West Road, Haidian District, Beijing 100094, China; fanlinkong@cau.edu.cn (F.K.); b20213040351@cau.edu.cn (S.W.); wei.wang@cau.edu.cn (W.W.); 2 Laboratory of Anatomy of Domestic Animals, Department of Basic Veterinary Medicine, College of Veterinary Medicine, China Agricultural University, No. 2 Yuanmingyuan West Road, Haidian District, Beijing 100094, China; BS20193050473@cau.edu.cn; 3 Microbial Biological Engineering Company Limited, Fanhua Road Jingkai District, Hefei 230009, China; caozan1314@126.com; 4 Beijing JingWa Agricultural Science and Technology Innovation Center, Mishan Road, Pinggu District, Beijing 101200, China; 13611235024@163.com (Y.L.); haoyangyi0928@cau.edu.cn (Z.Z.)

**Keywords:** *Acremonium terricola* culture, dairy cow, antioxidant capacity, milk performance, bacterial composition

## Abstract

*Acremonium terricola* culture (ATC) has similar bioactive constituents to *Cordyceps* and is known for its nutrient and pharmacological value, indicating the potential of ATC as a new feed additive in dairy cow feeding. The primary aim of this experiment was to investigate the effects of increasing amounts of ATC in diets on milk performance, antioxidant capacity, and rumen fermentation, and the secondary aim was to evaluate the potential effects of high doses of ATC. A total of 60 multiparous Holstein cows (110 ± 21 days in milk; 2.53 ± 0.82 parity) were assigned into 15 blocks and randomly assigned to one of four groups: 0, 30, 60, or 300 g/d of ATC per cow for 97 days. Data were analyzed using repeated measures in the Mixed procedure. Dry-matter intake was not changed (*p* > 0.05), while energy-corrected milk and fat-corrected milk yields increased linearly and quadratically, and somatic cell count in milk decreased linearly and quadratically (*p <* 0.05). The lactation efficiency and the yields of milk fat and protein increased linearly (*p* < 0.05). On day 90, serum catalase level, total oxidative capacity, glutathione peroxidase, immunoglobulin A, and immunoglobulin M concentrations were significantly higher in the 60 and 300 g/d groups than in the 0 g/d group (*p* < 0.05). ATC addition showed linear effects on total volatile fatty acid (VFA), acetate, branched VFA concentrations, and rumen pH (*p* < 0.05). Supplementing 60 and 300 g/d ATC significantly affected the bacterial composition (*p* < 0.05). The relative abundance of *Christensenellaceae*_*R–7_group* and *Lachnospiraceae*_*NK3A20_group* were significantly increased by 60 g/d supplementation, and the relative abundance of *Erysipelotrichaceae_UCG_002*, *Acetitomaculum*, *Olsenella*, and *Syntrophococcus* were significantly increased by 300 g/d supplementation (*p* < 0.05). ATC was effective in enhancing rumen fermentation and reducing somatic cell count in milk, thereby improving milk yield. The optimized dose of ATC was 60 g/d for lactating cows, and there were no risks associated with high doses of ATC.

## 1. Introduction

The health and biological function of livestock often have been prioritized in recent years [1]. Nutritional strategies have emerged, and they have been proposed as a key factor to improve the health status and welfare of animals, as well as to enhance productivity of livestock [2,3,4]. Feed additives, which are nutrients that when added to the feed can trigger the desired response of the animal’s body on production parameters, have been widely used in animal nutrition for quite a long time [5,6,7,8]. The main compounds used for this purpose are fermented feed, probiotics, prebiotics, synbiotics, or various types of residues from plant production. *Cordyceps* has a long history, and has been used for the last 300 years [9]. It is a rare, naturally entomopathogenic fungus that is mostly collected on the Himalayan plateau in China. Although wild *Cordyceps* has many functional bioactive ingredients, including cordycepin, cordycepic acid, and cordyceps polysaccharide, it takes a long time to grow under strict conditions, cannot be cultivated artificially, and can only be harvested once [10]. In recent decades, many studies have shown that the major functional components in *Cordyceps* possess multiple resistances to viruses, inflammation, oxidants, tumors, diabetes, and thrombi [9,11,12,13] Thus, many investigators have been devoted to artificial cultivation to obtain prebiotics [14,15].

*Acremonium terricola* is a parasite isolated from *Cordyceps gunnii*; artificial solid fermentation is then used to obtain *Acremonium terricola* culture (ATC). ATC is a new feed additive with bioactive ingredients that are the same as those of *C. gunnii*, such as *D*-mannitol, galactomannan, cordycepin, and essential amino acids [16]. As reported in previous studies, ATC supplementation enhanced the growth performance, antioxidant capacity, and immune function of calves [17]. ATC supplementation increased the total tract apparent digestibility and milk performance in dairy cows, then decreased somatic cell counts in milk [18,19]. Dairy cows during the transition period are at risk of diseases, and undergo extensive tissue mobilization due to a negative energy balance [20,21,22]. Li et al. [23] found that 30 g/d ATC ameliorated the negative energy balance and improved milk production and antioxidant capacity. These studies indicated that ATC was an effective feed additive for dairy cows at 30 g/d, and the maximum addition amount (30 g/d) was more effective than other additional amounts. However, studies on the effects of the higher dose of ATC supplementation on milk production are lacking, which means that the appropriate level of ATC in the diet is still not clear. In this regard, galactomannans from ATC are water-soluble polysaccharide polymers, and several studies have reported that galactomannan plays a crucial role in manipulating gastrointestinal microbiota by providing nutrients for beneficial microbes [12,24,25]. Additionally, D-mannitol can also become the sole energy source for bacterial growth in the rumen [26]. It is known that highly dense and diverse microbial populations in the rumen cause many differences in feeding modes and physiology between ruminants and monogastric animals [27]. Thus, ATC supplementation in dairy cows may have the potential to further improve milk performance and rumen function.

The ingredients and bioactivities of ATC are the same as those of wild *Cordyceps*. Owing to the wild occurrence, natural *Cordyceps* is regarded as pharmacologically secure. However, dry mouth, nausea, abdominal distension, throat discomfort, headache, diarrhea, and allergic reactions have been reported in *Cordyceps* studies [28,29,30]. Tests of cordycepin in dogs involved gastrointestinal toxicity and bone marrow toxicity [31]. However, these toxicity tests were conducted using monogastric animals instead of ruminants. Thus, future in-depth evaluation of the security of ATC will benefit its mass production and use as a feed additive.

We hypothesized that ATC supplementation would improve the milk performance, antioxidant capacity, and immune function of midlactating dairy cows, and reshape their microbiota. Hence, this study investigated the effects of different amounts of ATC on feeding behavior, ruminal fermentation, bacterial composition, serum variables, and milk performance, as well as the possible adverse effects of high doses of ATC, on lactating dairy cows.

## 2. Materials and Methods

### 2.1. Animals and Treatments

On a commercial dairy farm (Jinyindao Dairy Farm; Sanyuan Food Co., Ltd., Beijing, China), 60 multiparous lactating dairy cows averaging 110 ± 21 days (mean ± SD) in milk, 28 ± 3 kg/d of milk, 2.53 ± 0.82 parity, and 630 ± 50 kg of body weight were selected and assigned into 15 blocks according to days in milk (DIM) and milk yield, and then randomly allocated to 1 of 4 groups: 0, 30, 60, or 300 g/d of ATC for each cow, added into a total mixed ration (TMR). The ATC was provided by Microbial Biological Engineering Ltd. (Hefei, China), and the functional composition of the ATC is listed in Supplementary Table S1. The recommended dose for ATC was 30 g/d [19,32]. In this experiment, we included double the recommended dose to determine whether ATC had a linear and quadratic effect on lactation performance, as well as 10 times the recommended dose to test the potential effects on dairy cow health. The same barn equipped with water bowls and automatic weighing feeding equipment (Insentec B.V., Marknesse, The Netherlands) were provided for these cows to obtain individual feed intake. All cows were fed and milked 3 times per day at 09:00, 16:00, and 22:00. The ATC was top-dressed onto a TMR; ingredients and nutrient contents of the TMR are listed in Supplementary Table S2. The diet was formatted using National Research Council (NRC, 2001) [33] to meet the nutrient requirements of lactating dairy cows. The experiment period was 97 days, including 7 days to allow for adaptation, during which ATC was supplemented gradually. According to the standard operating procedure on this commercial dairy farm, all cows had de-worming (ivermectin, Harbin Qianhe Animal Medicine Manufacturing Co., Ltd., Harbin, China) during the dry-off period, and the farm followed the epidemic prevention and control procedures. All experimental procedures were submitted to the experimental animal welfare and animal ethics committee of China Agricultural University and approved (CAU2021009-2).

### 2.2. Sampling and Analysis

Individual feed intake was recorded daily using feed-weighing equipment (RIC system, Insentec B.V., Marknesse, The Netherlands), and the samples of TMR and orts were collected on the same day (the first day of every week). The TMR, orts, and ATC were used to determine the nutrient composition, including dry matter (DM, 105 °C for 5 h), acid detergent fiber (ADF), neutral detergent fiber (NDF), and crude protein (CP) contents [34,35]. Daily dry-matter intake (DMI) was calculated using Equation (1):

(1) DMI=as fed TMR × dry matter content − ort × dry matter content

The amino acid (AA) concentrations were analyzed with a Hitachi L-8900 automatic AA analyzer (HITACHI High-Tech Corporation Ltd., Shanghai, China). The concentrations of *D*-mannitol, galactomannan, 3’-deoxyadenosine, and ergosterol were determined using liquid chromatography [36].

Milk yield was recorded electronically, and 50 mL of representative milk from each cow was collected at 0, 30, 60, and 90 days using a diverter (BouMatic Company, Madison, WI, USA). The 50 mL milk sample included 20 mL of milk from the 09:00 milking, 15 mL from the 16:00 milking, and 15 mL from the 22:00 milking. Milk samples were stored at 4 °C for further analysis of milk composition (protein, fat, lactose, urea nitrogen, and somatic cell count) using a FOSS MilkoScanTM 7 (FOSS, Hillerod, Denmark).

Blood samples were collected from the tail vein of each cow into 5 mL Vacutainer K2EDTA tubes (OLABO Biotechnology Co., Ltd., Jinan, China) at 0, 45, and 90 days after morning feeding between 11:00 and 12:00. Blood serum was separated by centrifugation (3000× *g* for 10 min at 4 °C) after sampling immediately and stored at −20 °C. At the end of the experiment, all serum samples were analyzed using a GF-D200 Auto Analyzer (Caihong Analytical Instrument Co., Ltd., Gaomi, China) to determine urea nitrogen (SUN) and total amino acid (TAA) concentrations using standard commercial kits (BioSino BioTechnology and Science Inc., Beijing, China). The superoxide dismutase (SOD), catalase (CAT), malondialdehyde (MDA), total oxidative capacity (TAC), and glutathione peroxidase (GSH-Px) levels were determined by colorimetry using commercial kits (Nanjing Jian Cheng Bioengineering Institute, Nanjing, China). Immunoglobulin A (IgA), immunoglobulin G (IgG), and immunoglobulin M (IgM) concentrations were determined with ELISA kits (Abcam, Cambridge, UK) using a Thermo Multiskan Ascent (Thermo Fisher Scientific, Shanghai, China). The intra- and interassay coefficients of variations were both <10%.

Ruminal fluid was collected using an oral stomach tube after 2 h morning feeding from six cows at 0 and 90 days in each group, which accounted for close to the average milk production. The tube was cleaned thoroughly with fresh warm water after each sampling. The first 150 mL of rumen fluid was useless, and subsequent rumen fluid was collected and filtered through four layers of cheesecloth to obtain the fluid fraction. The fluid fraction pH value was measured with a Sartorius PB-10 pH meter (Beijing Sartorius Instrument Systems Co., Ltd., Beijing, China). The rumen fluid was stored in a 20 mL centrifuge tube, then frozen at –20 °C for volatile fatty acid (VFA) analysis. The rest of the fluid was stored in a 2 mL centrifuge tube and frozen at –80 °C for 16S sequencing. VFA content was quantified using gas chromatography (Agilent 6890N, Agilent Technology, Inc., Beijing, China), according to Kong et al [37].

Total DNA was extracted from 1 mL rumen fluid samples (thawing for 10 min) using an E.Z.N.A.^®^ Soil DNA Kit (Omega Bio-Tek, Norcross, GA, USA) according to the manufacturer’s instructions. The DNA extract was analyzed on a 1% agarose gel, and DNA concentration and purity were determined using a NanoDrop 2000 UV–vis spectrophotometer (Thermo Scientific, Wilmington, DE, USA). The hypervariable region V3-V4 of the bacterial 16S rRNA gene was amplified with primer pairs 338F (5′-ACTCCTACGGGAGGCAGCAG-3’) and 806R (5′–GGACTACHVGGGTWTCTAAT-3′) using an ABI GeneAmp^®^ 9700 PCR thermocycler (ABI, Foster City, CA, USA) [38]. The PCR amplification of the 16S rRNA gene was performed according to Kong et al [38]. The PCR product was extracted from a 2% agarose gel and purified using an AxyPrep DNA Gel Extraction Kit (Axygen Biosciences, Union City, CA, USA) according to the manufacturer’s instructions, and quantified using a Quantus™ Fluorometer (Promega, Madison, WI, USA). Purified amplicons were pooled in equimolar amounts and paired-end sequenced on an Illumina MiSeq PE300 platform (Illumina, San Diego, CA, USA).

The raw 16S rRNA gene sequencing reads were demultiplexed, quality-filtered using Fastp (Version 0.20.1, Haplox, Shenzhen, China) [39], and merged using FLASH version 1.2.7 (The Center for Computational Biology at Johns Hopkins University, MD, USA) [40] with the criteria set according to Huang et al [41]. All samples were subsampled to equal size of 25,122 sequences for downstream analysis. Operational taxonomic units (OTUs) with a 97% similarity cutoff were clustered using UPARSE version 7.1 (Independent Investigator, CA, USA) [42], and chimeric sequences were identified and removed. The taxonomy of each OTU representative sequence was analyzed using the RDP Classifier version 2.2 (Center for Microbial Ecology, Michigan State University, MI, USA) [43] against the 16S rRNA database (e.g., Silva v138) using a confidence threshold of 0.7 [44]. The relative abundance of phylum or genus ≥ 1% was used to consider the predominant phylum or genus. Alpha diversity indices (observed richness, Chao1, ACE, Shannon, Simpson) and Good’s coverage were obtained using the alpha rarefaction script in QIIME [45]. Principal coordinates analysis (PCoA) was conducted by Bray–Curtis matrices in R (http://www.rstudio.com, accessed on 3 May 2021). An analysis of similarities (ANOSIM) using Bray–Curtis distance matrices was performed to test the statistical differences among the observed microbial profiles.

### 2.3. Statistical Analysis

The effects on feeding behavior, milk performance, serum variables, and rumen fermentation parameters were analyzed using the Mixed procedure in Statistical Analysis System 9.4 (SAS Institute Inc., Cary, NC, USA). Before analyses, data were screened for normality using the Univariate procedure; the variables except for sequencing data met the assumptions for normality. A randomized block experimental design was used for the month, treatment, and interaction of treatment and month according to the following model:

(2) $$Y_{\mathrm{ijkl}}=\mu +A_{i}{+T}_{j}{+\mathrm{AT}}_{\mathrm{ij}}{+B}_{k}{+\varepsilon }_{\mathrm{ijkl}}$$

where Y is the dependent variable, μ is the overall mean, A_i_ is the fixed effect of ATC supplementation, T_j_ is the repeated effect, AT_ij_ is the interaction effect of A and T, B_k_ is the block effect, and ε_ijkl_ is the random residual error. Cows were used as the experimental units. The linear and quadratic effects of the treatment on these variables were tested using orthogonal polynomials, accounting for unequal spacing of ATC supplementation levels. The results are presented as least-squares means and were separated using the PDIFF statement when the fixed effects were significant. Significance was declared at *p* ≤ 0.05, and tendencies were declared at 0.05 ≤ *p* ≤ 0.10.

The effects on alpha diversity indices and the relative abundance of genera were assessed using the Kruskal–Wallis H test, and Dunn’s test was applied to conduct multiple comparisons. All *p*-values were corrected using a false discovery rate of 0.05, and a corrected *p*-value < 0.05 was considered significant. The data are presented as the mean ± standard error (SEM). Spearman’s correlations were calculated between the relative abundances of genera. Significant correlations were defined as −0.7 > *r* > 0.7, and *p* < 0.05.

## 3. Results

### 3.1. Feeding Behavior

Table 1 shows the effects of ATC supplementation on feeding behavior. In the third month, 30 g/d ATC supplementation increased DMI (*p* < 0.05), whereas DMI/feeding frequency, average DMI, and average feeding frequency were not changed after ATC supplementation (*p* > 0.05). The different levels of ATC supplementation decreased feeding duration and feeding duration/feeding frequency, and increased DMI/feeding duration linearly (*p* < 0.05).

### 3.2. Milk Performance

As shown in Table 2, the performances including milk yield, fat-corrected milk yield (FCM), and energy-corrected milk yield (ECM) increased linearly and quadratically with ATC supplementation level (*p* < 0.05). There were no significant differences between the average milk yield and average ECM yield between the 60 g/d group and 300 g/d groups (*p* > 0.05), which were both higher than those of cows in the 30 g/d and 0 g/d groups (*p* < 0.05). The milk efficiency values both increased linearly with ATC supplementation level (*p* < 0.05), while there was no difference between the 60 g/d ATC treatment and 300 g/d ATC treatment groups (*p* > 0.05).

Table 3 shows the effects of different amounts of ATC supplementation on milk composition. Milk protein, milk fat, milk lactose, and milk urea nitrogen (MUN) were not affected by ATC supplementation (*p* > 0.05). Milk protein yield, milk fat yield, and somatic cell count (SCC) were linearly influenced by ATC supplementation (*p* < 0.05). The milk protein yield in the first month was not increased after ATC supplementation (*p* > 0.05). The milk protein yield in the second and third months and the average yield were increased after 60 g/d and 300 g/d ATC supplementation (*p* < 0.05), but the milk protein yield and milk fat yield did not differ between the 60 g/d and 300 g/d ATC supplementation groups (*p* > 0.05). The SCC decreased in both a linear and quadratic manner after supplementation with ATC (*p* < 0.05), but there was no significant difference between the 60 g/d and 300 g/d ATC supplementation groups (*p* > 0.05).

### 3.3. Serum Variables

**Figure 1:**
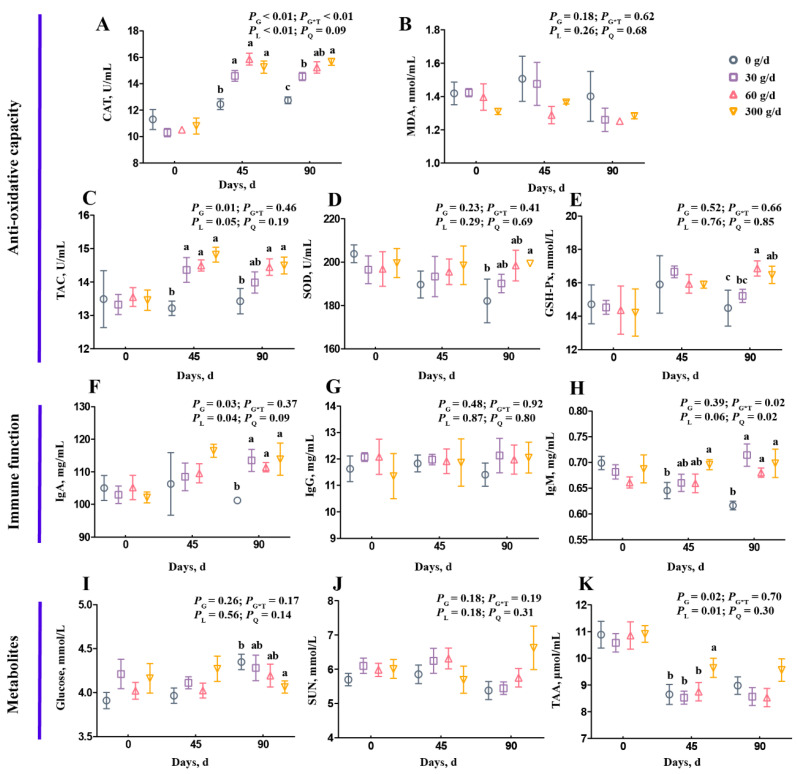
Effects of different amounts of *Acremonium terricola* culture on serum variables in lactating dairy cows. The serum variables were divided into three groups, including antioxidant capacity (**A**–**E**), immune function (**F**–**H**), and metabolites (**I**–**K**). Serum variables of dairy cows fed a diet with supplementation of ATC at 0, 30, 60, or 300 g/d were used. The effects included group effect and the interaction effect of group and time, as well as linear and quadratic effects. Different lowercase letters denote significant differences among treatments (*p* < 0.05). ATC, *Acremonium terricola* culture; CAT, catalase; MDA, malonaldehyde; TAC, total oxidative capacity; GSH–Px, glutathione peroxidase; SOD, superoxide dismutase; IgA, immunoglobulin A; IgG, immunoglobulin G; IgM, immunoglobulin M; SUN, serum urea nitrogen; TAA, total amino acids. Mean ± SEM. *n* = 15.

Figure 1 shows the summary of the statistics for serum variables, including antioxidant capacity, immune function, and metabolites. On day 0, there was no significant difference on any serum variables among the four groups (Figure 1; *p* > 0.05). For antioxidant capacity, the CAT and TAC concentrations increased linearly after ATC supplementation (Figure 1A,C; *p* < 0.05). On day 90, the 60 g/d and 300 g/d ATC supplementation increased CAT, TAC, and GSH-Px concentrations (Figure 1A,C,E; *p* < 0.05). Only 300 g/d ATC supplementation increased SOD concentration on day 90 (Figure 1D; *p* < 0.05). For immune function, the ATC supplementation did not influence IgG concentration (Figure 1G; *p* > 0.05), but significantly increased IgA and IgM concentrations on day 90 (Figure 1F,H; *p* < 0.05). The IgA concentration increased linearly, and the IgM concentration increased quadratically after ATC supplementation (Figure 1F,H; *p* < 0.05). However, there were no differences among the 30 g/d, 60 g/d, and 300 g/d supplementation groups on day 45 and day 90 (Figure 1F,H; *p* > 0.05). For metabolites in the serum, the glucose concentration of the 300 g/d group was lower than in the 0 g/d group on day 45 (Figure 1I; *p* < 0.05). The SUN concentration was not affected by ATC supplementation (Figure 1J; *p* > 0.05). The TAA concentration increased linearly after ATC supplementation (Figure 1K; *p* < 0.05), and was higher in 300 g/d group on day 45 than in the 0, 30, and 60 g/d groups (Figure 1K; *p* < 0.05).

### 3.4. Rumen Fermentation Parameters

**Figure 2:**
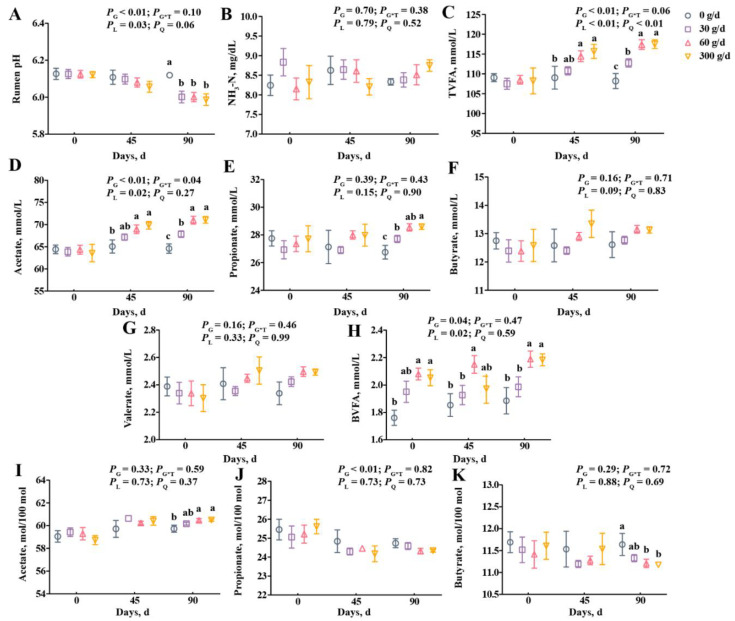
Effects of different amounts of *Acremonium terricola* culture on rumen pH (**A**), NH_3_-N (**B**), and volatile fatty acids (**C**–**K**) in lactating dairy cows. The VFA are expressed as concentration (**C**–**H**) and molar proportion (**I**–**K**). The effects included group effect and the interaction effect of group and time, as well as linear and quadratic effects. Different lowercase letters denote significant differences among treatments (*p* < 0.05). ATC, *Acremonium terricola* culture; TVFA, total volatile fatty acid; BVFA, branched volatile fatty acid; Mean ± SEM. *n* = 15.

From the data in Figure 2, we observed a linear decrease in the rumen pH, and the pH of the 0 g/d group was higher than in other groups on day 90 (Figure 2A; *p* < 0.05). The supplementation did not affect ruminal NH_3_-N, propionate, butyrate, or valerate concentrations (Figure 2B,E–G; *p* > 0.05). Treatment significantly affected the molar proportion of propionate with no linear or quadratic effects (Figure 2J), and the molar proportion of butyrate decreased after 60 g/d and 300 g/d supplementation at day 90 (Figure 2K). The total volatile fatty acid (TVFA), acetate, and branched volatile fatty acid (BVFA) concentrations increased linearly with ATC supplementation (Figure 2C,D,H; *p* < 0.05), but the molar proportion of acetate only significantly increased after 60 g/d and 300 g/d supplementation at day 90 compared with that of the 0 g/d group (Figure 2I; *p* < 0.05). The TVFA and acetate concentrations were higher in cows fed with 60 g/d and 300 g/d of ATC at day 45, and were higher in the 30, 60, and 300 g/d groups on day 90 than that of those fed with 0 g/d of ATC (Figure 2C,D; *p* < 0.05).

### 3.5. Diversity of Rumen Microbiota

**Figure 3:**
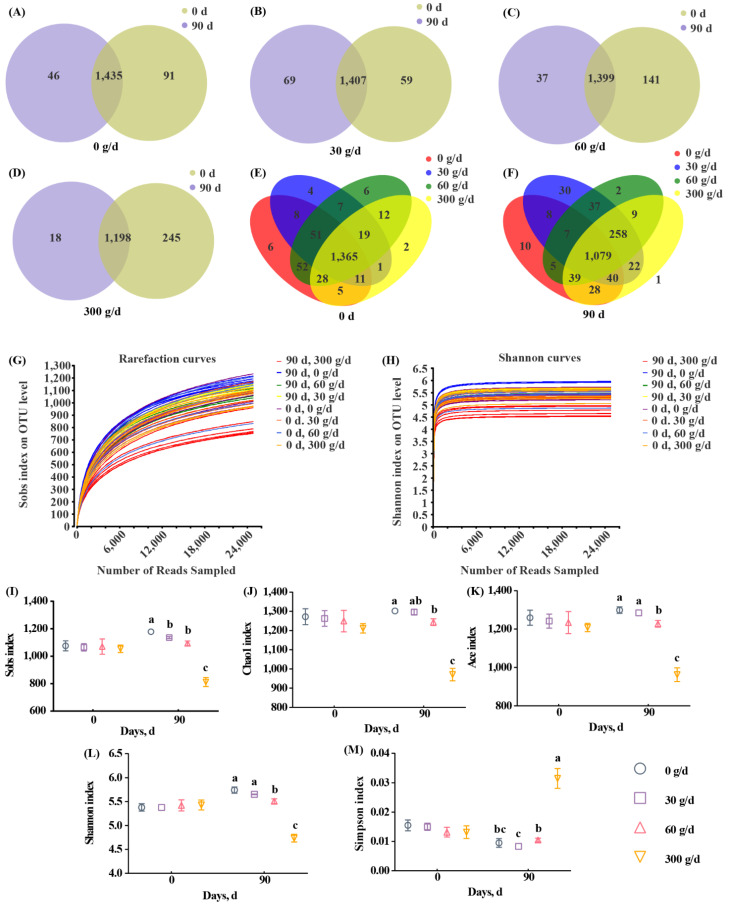
OTU number and alpha diversity responses of the ruminal microbiota to *Acremonium terricola* culture supplementation in lactating dairy cows. (**A**–**F**) Venn diagrams showing the number of OTUs in the 0 g/d (**A**), 30 g/d (**B**), 60 g/d (**C**), and 300 g/d (**D**) groups at day 0 (**E**) and day 90 (**F**); (**G**) Shannon curves; (**H**) rarefaction curves; (**I**–**M**) alpha diversity. Different lowercase letters denote significant differences among treatments (Figure 3I–M, *p* < 0.05). OTU, operational taxonomic unit. Mean ± SEM. *n* = 6.

Next, we assessed the diversity of rumen microbiota. The range for Good’s coverage was 99.05–99.25%, indicating a good sequencing depth (Supplementary Table S3). The 16S rRNA sequencing showed a total of 1583 OTUs across all samples, with 97% similarity (Figure 3A–F). Rarefaction curves and Shannon curves showed a decreased number of new OTUs and Shannon index as the sequencing number increased (Figure 3G,H). A total of 1435, 1407, 1399, and 1198 OTUs were shared between day 0 and day 90 in the 0, 30, 60, and 300 g/d groups, respectively (Figure 3A–D). A total of 1365 and 1079 OTUs were shared among the four groups on day 0 and day 90, respectively. The alpha diversity analysis shows that there were no significant differences among these groups at day 0 (Figure 3I–M). At day 90, 300 g/d ATC supplementation significantly decreased Sobs, Chao1, ACE, and Shannon indices, and significantly increased the Simpson index when compared with those of other groups (Figure 3I–M; *p* < 0.05). There were significant differences between the 60 g/d group and the 0 g/d group with regards to alpha diversity indices, except for the Simpson index (Figure 3I–M; *p* < 0.05). The 30 g/d ATC supplementation only significantly decreased the Sobs index when compared with that of the 0 g/d group (Figure 3I; *p* < 0.05).

**Figure 4:**
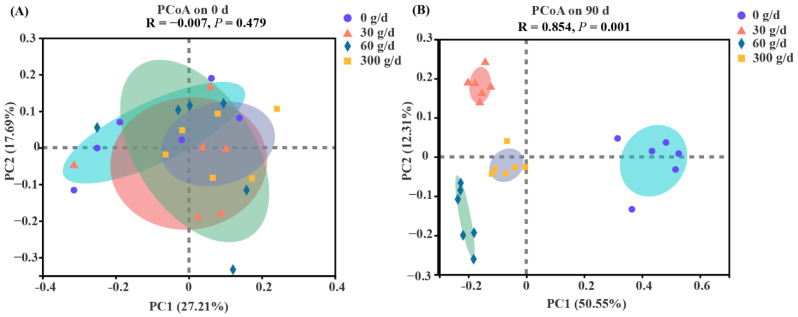
Bate diversity responses of the rumen microbiota to *Acremonium terricola* culture supplementation in lactating dairy cows on day 0 (**A**) and day 90 (**B**). Rumen microbiota of dairy cows fed a diet with supplementation of ATC at 0, 30, 60, or 300 g/d were used. Analysis of similarities (ANOSIM) using Bray–Curtis distance matrices was used to test the statistical differences. *n* = 6.

The PCoA was conducted based on the OTU level with ANOSIM analysis to test the statistical differences among the groups (Figure 4). No clear separation was found for rumen bacteria at day 0 (*p* > 0.05). The results showed distinct clustering according to the amount of ATC at day 90 (*p* < 0.05).

### 3.6. Microbial Profiles of Rumen Microbiota

**Figure 5:**
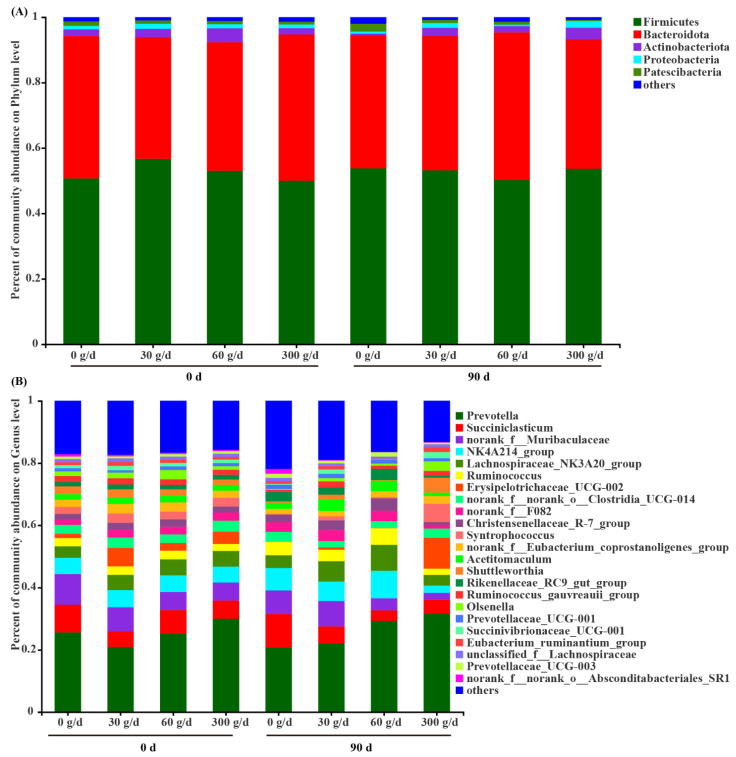
Composition of the rumen microbiota at the phylum level (**A**) and genus level (**B**) before (day 0) and after (day 90) of *Acremonium terricola* culture supplementation in lactating dairy cows. The relative abundance of taxa ≤ 0.01% belonged to others. *n* = 6.

Among the phyla detected in the rumen (Figure 5A), Firmicutes (52.65 ± 1.17), Bacteroidota (41.39 ± 1.24), Actinobacteriota (2.41 ± 0.40), Proteobacteria (1.20 ± 0.20), and Patescibacteria (1.11 ± 0.10) were predominant, followed by Spirochaetota (0.57 ± 0.05). The predominant genera (Figure 4B) were *Prevotella* (25.91 ± 1.94), *NK4A214_group* (6.18 ± 0.58), *Succiniclasticum* (6.04 ± 0.84), *Lachnospiraceae_NK3A20_group* (5.59 ± 0.52), *Ruminococcus* (3.83 ± 0.39), *Erysipelotrichaceae_UCG–002* (2.69 ± 0.95), *Christensenellaceae_R–7_group* (2.65 ± 0.28), *Acetitomaculum* (2.58 ± 0.32), *Rikenellaceae_RC9_gut_group* (2.44 ± 0.27), *Syntrophococcus* (1.92 ± 0.61), *Shuttleworthia* (1.73 ± 0.47), *Ruminococcus_gauvreauii_group* (1.28 ± 0.19), *Prevotellaceae_UCG–001* (1.26 ± 0.08), *Olsenella* (1.25 ± 0.37), and *Prevotellaceae_UCG–003* (1.02 ± 0.10). Notably, 14.87% of reads recovered from the rumen could not be confidently assigned at the genus level.

### 3.7. Changes in the Rumen Bacterial Composition with Acremonium terricola Culture Supplementation

**Figure 6:**
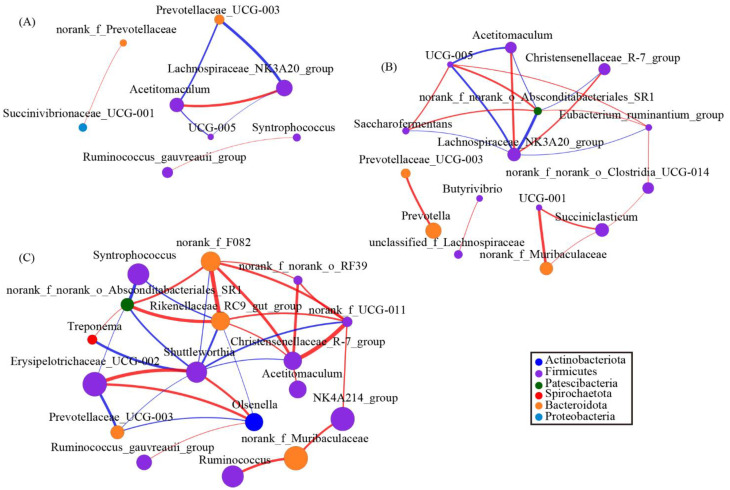
The network of co-occurring genera within the rumen microbiota supplied with different amounts of *Acremonium terricola* culture: (**A**) 0 and 30 g/d; (**B**) 0 and 60 g/d; (**C**) 0 and 300 g/d. The figure shows genera based on Spearman’s correlation. The nodes represent the core genera, and the size of each node is proportional to the degree. The edges stand for strong (Spearman’s correlation coefficient r > 0.7 or r < −0.7) and significant (*p* < 0.05) correlations between core genera. The nodes are colored based on phylum. Red and blue lines represent positive and negative correlations between two nodes, respectively.

To explore the interactions within genera, we used network analysis based on strong and significant correlations of core genera between the 0 g/d group and ATC-treatment groups. The network, including both 0 g/d and 30 g/d groups (Figure 6A), consisted of eight nodes (core genera) and seven edges (relations). *Acetitomaculum* and *Lachnospiraceae_NK3A20_group* had positive correlations, and *Syntrophococcus* and *Ruminococcus_gauvreauii_group* also had positive correlations (Figure 6A). Similar to the network mentioned above, the network analysis from 0 g/d and 60 g/d showed that *Lachnospiraceae_NK3A20_group* had a positive correlation with *Acetitomaculum* and *Christensenellaceae_R–7_group* (Figure 6B). This network consisted of 15 nodes and 19 edges, whereas 11 of the 15 nodes belonged to Firmicutes (Figure 6B). The last network of 0 g/d and 300 g/d showed 17 nodes and 33 edges. *Acetitomaculum* and *Christensenellaceae_R–7_group* were also found in the networks (Figure 6C). Furthermore, 10 of the 17 nodes belonged to Firmicutes (Figure 6C). The genera *Syntrophococcus*, *Shuttleworthia*, *Erysipelotrichaceae_UCG–003*, and *Olsenella* were part of the network (Figure 6C).

**Figure 7:**
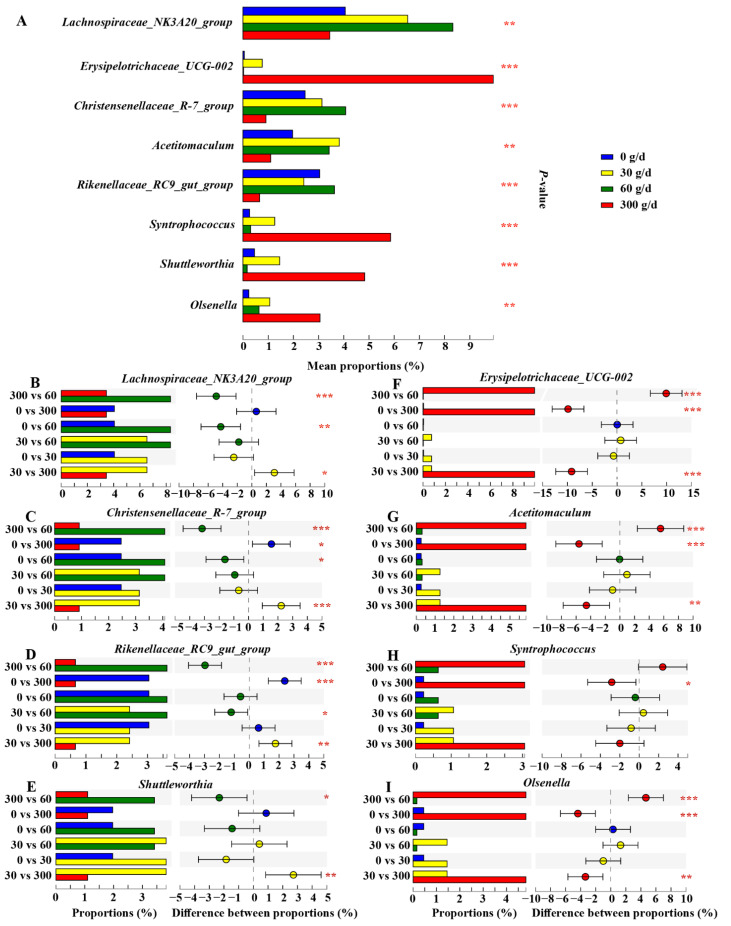
Effects of different levels of *Acremonium terricola* culture supplementation on the relative abundance of ruminal core genera (≥1%) in lactating dairy cows. The figure only shows the significantly different genera. The Kruskal–Wallis H test was used. *, 0.01 < *p* ≤ 0.05; **, 0.001 < *p* ≤ 0.01; ***, *p* ≤ 0.001. *n* = 6.

We then conducted the Kruskal–Wallis H test to filter significantly different core genera (relative abundance ≥ 1%), as shown in Figure 7. The results showed that the relative abundance of *Lachnospiraceae_NK3A20_group* and *Christensenellaceae_R–7_group* increased after 60 g/d supplementation compared to that in the 0 g/d group (*p* > 0.05). The cows fed 300 g/d ATC had a lower relative abundance of *Lachnospiraceae_NK3A20_group*, *Christensenellaceae_R–7_group*, *Rilkenellaceae_RC9_gut_group*, and *Shuttleworthia* than that of cows fed 30 g/d and 60 g/d (*p* < 0.05), while there was a higher relative abundance of *Erysipelotrichaceae_UCG_002*, *Acetitomaculum*, and *Olsenella* than that of cows fed 0, 30, and 60 g/d (*p* > 0.05). The relative abundance of *Syntrophococcus* was also significantly increased in the 300 g/d group compared to that in the 0 g/d group (*p* > 0.05).

## 4. Discussion

ATC is a new type of new feed additive with functional components similar to those of *Cordyceps*. The positive effects of ATC on livestock production and health have been reported in rats [16], calves [17], and dairy cows [19]. However, their pharmacological and biochemical actions, particularly in applications in ruminants, have not been clearly elucidated. In our study, DMI was not affected, regardless of the ATC dosage. Individual DMI is critical, because insufficient intake represents limited milk synthesis. The unaltered DMI agreed with the results of previous studies showing that ATC or *Cordyceps* spp. supplementation improved body weight and feed efficiency rather than DMI [17,19,46,47]. However, we noted differences in feeding behavior. Dairy cows treated with ATC had a shorter feeding duration. Although lying time and rumination time were not measured in this study, this result implied that the dairy cows might have a longer time to lie and ruminate after feeding. Increased rumination time and lying time have long been associated with increased milk performance, with the general belief that rumination increases the surface area of feed particles, making them more accessible to microbes [48], and increased lying time is associated with decreased lameness [49]. This is in agreement with the higher milk performance observed in this study.

Consistent with the report of Li et al. [32], we observed that supplemental ATC improved milk yield linearly without influencing DMI, resulting in improved milk efficiency. Although maximum yields of milk and milk efficiency were obtained at 300 g/d ATC, increasing the dosage from 60 g/d to 300 g/d had a limited positive effect on milk performance. Many factors may have contributed to this response. Lactose yield determines the amount of absorbed water in the alveoli, and thus, the volume of the produced milk [50]. Approximately 20% of the circulating blood glucose in dairy cows is converted into lactose [50]. Thus, it is not surprising that ATC supplementation increased milk lactose yield. Moreover, Costa et al. [51] demonstrated that SCC was negatively correlated with milk yield and milk lactose percentage. SCC is a well-established indicator of mammary gland inflammation, which is highly correlated with the presence of a mammary infection. The dairy cows used in our study had less than 150,000 cells/mL SCC among all treatments, which was considered healthy, and had low clinical mastitis incidence [52]. The SCC in milk decreased quadratically in cows fed different levels of supplemental ATC, with the highest decrease in cows fed ATC at 300 g/d. Many studies have demonstrated that cordycepin and galactomannan in *Cordyceps* or ATC functioned as antioxidants. Li et al. [16] indicated that ATC played anti-inflammatory and antioxidant roles by inhibiting the mitogen-activated protein kinase signaling pathway, and ATC increased the TAC and GSH-Px concentrations in dairy cows when fed up to 30 g/d [32]. Waewaree et al. [53] also indicated that weaned pigs fed a diet supplemented with the spent mushroom compost of *Cordyceps militaris* displayed greater immunoglobulin secretion, lower inflammation, and a lower pathogenic population. These data indicated that ATC had positive effects on immune and antioxidant functions, which was consistent with the serum variables and SCC changes in our study. We speculated that a possible explanation for our results was that ATC improved the immune function and antioxidant capacity of cows, thereby decreasing SCC and improving milk yield. Moreover, when cows were fed with 60 g/d and 300 g/d of ATC, ECM and FCM yields were greater at 60–90 days than those in 0 g/d and 30 g/d, which might have been due to the adaptation periods of the cows to the ATC.

According to the CP concentration in ATC in our study, 30, 60, and 300 g/d ATC supplementation supplied a total of 7.36, 14.72, and 73.59 g/d CP for cows, respectively. Barros et al. [54] found that a linear response to increasing dietary CP concentration increased MUN concentration and milk protein yield, which was inconsistent with the unchanged MUN and SUN concentrations in our study. This discrepancy is potentially explained by the benefits of balancing more adequate amounts of AA in the diet for N efficiency [55]. SUN and MUN concentrations can be used as indicators of microbial protein synthesis and N efficiency. Some N-NH_3_ in the rumen, which is not used for microbial protein synthesis and absorbed from the rumen, would be used to produce urea in the liver, which consumes energy and reduces N utilization [56]. Thus, the unchanged N-NH_3_ concentration in the rumen indicated that extra N intake from ATC supplementation contributed to microbial protein synthesis, rather than N-NH_3_ production. Many studies have demonstrated that the supply of intestinally available limiting AA is pertinent to milk yield and milk protein yield by decreasing protein mobilization and increasing accretion [57,58]. According to the AA profile of ATC, TAA accounted for 17.27% of ATC (DM basis). Hence, under the current experimental conditions, ATC supplementation improved milk protein yield in a quadratic manner, possibly by reducing urea synthesis to save energy for protein synthesis and improve the balance of available AA in the mammary gland of lactating cows. Further studies on ruminal digestibility and intestinal availability of ingredients (cordycepin, D-mannitol, galactomannan, AA) of ATC will be useful in formulating an accurate ration.

Chen et al. [59] reviewed the toxicity of known metabolites identified from *Cordyceps* fungi and concluded that different compounds showed antimicrobial activity, and may have the potential to threaten the safety of the poultry industry. However, Ramos et al. [60] revealed that weaned pigs fed 1.5 g/kg of *Cordyceps militaris* spent mushroom compost showed reduced populations of pathogenic Gram-negative *E. coli* and increased populations of beneficial *Lactobacillus* spp. These different observations were partially attributed to the different species (over 1000) of *Cordyceps* fungi [59]. Hence, we evaluated the effects of ATC on the microbiota responses of dairy cows. No differences in bacterial diversity on day 0, and decreases in bacterial richness and evenness at day 90 after ATC treatment, were identified in this study, indicating that ATC supplementation depressed the microbiota. Furthermore, the common OTUs between day 0 and day 90 among these groups, as well as alpha diversity on day 90, changed with increasing levels of ATC supplementation, suggesting that ATC depressed the microbiota in a dose-dependent manner in dairy cows fed with increasing levels of ATC.

The pH and VFA concentrations are the indicators of rumen function and the ruminal environment. It has been reported that ATC increased CP digestibility of three types of roughage [19], and dairy cows fed ATC had an increased abundance of cellulolytic, proteolytic, and amylolytic bacteria in the rumen, which resulted in enhanced ruminal fermentation with increased VFA production and a decreased pH [32]. Unlike RT–PCR detection of selected bacterial species in a previous study [32], the changes in rumen microbiota in our study were analyzed using 16S gene sequencing. Although the microbiota was depressed, 30, 60, and 300 g/d ATC supplementation resulted in decreased pH and increased TVFA concentration. Reductions in the richness and diversity of microbiota in the rumen have been associated with higher production efficiency in dairy cattle [61]. According to the network of co-occurring genera and the Kruskal–Wallis H test, certain genera showed a strong and significant interaction with each other, and may contribute to increased milk performance. As the most abundant genera in the Firmicutes, *Lachnospiraceae_NK3A20_group* was characterized by cellulose-decomposing activity and starch hydrolysis, which could produce acetate and formate, among others [62]. The *Christensenellaceae_R–7_group*, belonging to the family Christensenellaceae, produces acetate and butyrate, with the ability to utilize arabinose, glucose, and mannose [63]. Galactomannan in ATC may support the growth of *Christensenellaceae_R–7_group*. *Acetitomaculum* is also an acetogenic genus that utilizes formate, glucose, and hydrogen to reduce methane production [64]. Finally, *Olsenella* ferments starch and glycogen substrates and produces lactic, acetic, and formic acids [65]. These findings corroborated our results, in which TVFA and acetate concentrations and molar proportions of acetate were increased. In truth, different taxa are associated with feed efficiency and methane emissions. Ramayo-Caldas et al. [66] identified a higher abundance of the Christensenellaceae and Lachnospiraceae families associated with methane production in dairy cows, and McLoughlin et al. [67] found that the genera *Olsenella* and *Acetitomaculum* exhibited negative associations with the feed conversion ratio and average daily gain, respectively. Our study was limited by the absence of methane emissions analysis, and further studies could provide more reliable estimates of the microbiota contribution to energy flow. Overall, although 30 g/d and 60 g/d ATC supplementation were linked to lower richness of microbiota, a simpler bacterial composition could result in increased dominance of specific functional components, which led to higher concentrations of products that were relevant to the dairy cows.

The extent of variability in OTU richness and diversity between groups was mostly associated with significant alterations in the microbiota [68,69]. The greatest changes in microbiota were obtained from the 0 g/d group to 300 g/d group, rather than the 30 g/d or 60 g/d groups. As described above, the abundance of *Lachnospiraceae_NK3A20_group*, *Christensenellaceae_R–7_group*, and *Acetitomaculum* was increased in the 30 g/d and 60 g/d groups, but decreased in the 300 g/d group, compared with that in the 0 g/d group, followed by that of *Rilkenellaceae_RC9_gut_group*. *Rilkenellaceae_RC9_gut_group* belongs to the family Rilkenellaceae, which are specialized for the tract environment, with anaerobic metabolisms, and the metabolic end-products (acetate, propionate, and succinic acid) are produced from glucose, lactose, and mannose [70]. Conversely, the decrease in the relative abundance of these genera may be attributed to high doses of cordycepin. A previous study found that just 30 g/d of ATC supplementation decreased the relative abundance of Fibrobacter succinogenes in the rumen of dairy cows, which are highly specialized in cellulose degradation [32]. This was similar to the findings of Huang et al. [71], who also observed cordycepin as an efficient killer of Mycobacterium tuberculosis and Mycobacterium bovis. Cordycepin showed antibacterial activity in these studies. Additionally, the interaction between protozoa and bacteria (influenced by ATC) also partly explained the depressed genera. The interaction of ciliates with other microbial groups in the rumen has been previously reported, documenting predation on other rumen microbes, such as bacteria, fungi, and protozoa as well [72]. Li et al. [32] reported that ATC supplementation improved the relative abundance of ciliate protozoa. Hence, we speculated that ATC reshaped the bacterial structure in the rumen of dairy cows by regulating ciliate protozoa. Moreover, we noted that inoculation of defaunated sheep with protozoa further promoted an increase in the abundance of *Syntrophococcus* [73], which agreed with our results.

The relative abundance of the three genera showed significant enrichment in the 300 g/d group; namely, *Erysipelotrichaceae_UCG_002*, *Syntrophococcus*, and *Shuttleworthia*, rather than in the 30 g/d or 60 g/d groups. *Erysipelotrichaceae_UCG_002* belongs to the Erysipelotrichaceae family and has been previously reported to be associated with VFA synthesis and energy generation. Hao et al. [74] indicated that *Erysipelotrichaceae_UCG_002*, *Syntrophococcus*, and *Shuttleworthia* were negatively correlated with rumen pH, and speculated that these genera were involved in fiber digestion. Acetate is the end product of fiber decomposition. Our results indicated that 300 g/d ATC supplementation changed the bacterial composition and enhanced fiber digestion differently compared with the 30 g/d or 60 g/d groups, and resulted in the same improvement of VFA production and modification of pH.

## 5. Conclusions

Feeding different levels of ATC to lactating dairy cows improved milk yield without affecting DMI, thus increasing milk protein yield. The improvement in milk yield was likely related to improved immune function and antioxidant capacity, which led to decreased SCC, or possibly was due to the improvement in rumen fermentation with a simpler bacterial composition, which favored more effective digestion to produce VFA. Under the current experimental conditions, the optimal dose of ATC supplementation was approximately 60 g/d. The 300 g/d high-dose ATC reshaped the microbiota differently without effects on milk performance when compared with 60 g/d ATC, suggesting that the microbiota responded differently to the individual active components in ATC at increased concentrations. Hence, experiments to test the effects of purified active components (e.g., cordycepin, *D*-mannitol, galactomannan) on microbiota are needed to provide more information on ATC as a feed additive.

## Figures and Tables

**Table 1:** Effect of different amounts of *Acremonium terricola* culture on feeding behavior in lactating dairy cows.

Items	ATC Supplementation, g/d per Head				SEM	*p*-Value			
	0	30	60	300		G	G × T	L	Q
DMI, kg/d									
Average	21.70	21.95	21.91	21.87	0.103	0.36	0.56	0.71	0.66
0–30 d	21.93	22.01	22.16	21.84	0.182	0.17			
30–60 d	21.38	21.52	21.59	21.68	0.170	0.21			
60–90 d	21.81 ^b^	22.32 ^a^	21.97 ^ab^	22.10 ^ab^	0.159	0.04			
Feeding frequency, bouts/d									
Average	20.33	20.71	21.65	21.15	0.540	0.27	0.32	0.35	0.90
0–30 d	19.85 ^b^	21.28 ^ab^	22.38 ^a^	20.28 ^ab^	0.884	0.04			
30–60 d	19.91	19.45	19.86	21.47	0.826	0.09			
60–90 d	21.24	21.41	22.70	21.70	0.769	0.22			
Feeding duration, h/d									
Average	4.40 ^a^	3.67 ^c^	3.57 ^c^	3.97 ^b^	0.038	<0.01	0.01	<0.01	<0.01
0–30 d	4.13 ^a^	3.56 ^b^	3.30 ^c^	3.60 ^b^	0.068	<0.01			
30–60 d	4.48 ^a^	3.64 ^c^	3.53 ^c^	3.96 ^b^	0.064	<0.01			
60–90 d	4.61 ^a^	3.80 ^c^	3.87 ^c^	4.34 ^b^	0.059	<0.01			
DMI/Feeding frequency, kg/bouts									
Average	1.08	1.07	1.03	1.04	0.026	0.37	0.32	0.50	0.83
0–30 d	1.13	1.05	1.02	1.10	0.043	0.06			
30–60 d	1.08	1.12	1.09	1.01	0.040	0.06			
60–90 d	1.03	1.05	0.97	1.02	0.037	0.16			
Feeding duration/Feeding frequency, min/bouts									
Average	13.07 ^a^	10.74 ^b^	10.01 ^c^	11.35 ^b^	0.261	<0.01	0.45	<0.01	0.20
0–30 d	12.70 ^a^	10.17 ^bc^	9.13 ^c^	10.90 ^b^	0.464	<0.01			
30–60 d	13.52 ^a^	11.32 ^b^	10.68 ^b^	11.12 ^b^	0.434	<0.01			
60–90 d	12.99 ^a^	10.73 ^b^	10.24 ^b^	12.04 ^a^	0.404	<0.01			
DMI/Feeding duration, kg/h									
Average	4.94 ^d^	6.01 ^b^	6.18 ^a^	5.55 ^c^	0.066	<0.01	0.01	<0.01	0.04
0–30 d	5.37 ^c^	6.20 ^b^	6.73 ^a^	6.08 ^b^	0.107	<0.01			
30–60 d	4.75 ^c^	5.93 ^a^	6.12 ^a^	5.48 ^b^	0.100	<0.01			
60–90 d	4.71 ^c^	5.89 ^a^	5.70 ^a^	5.10 ^b^	0.093	<0.01			

ATC, *Acremonium terricola* culture; SEM, standard error; DMI, dry-matter intake. The effects included group effect (G) and the interaction effect of group and time (G × T), as well as linear (L) and quadratic effects (Q). Different lowercase letters in the same row show significant differences (*p* < 0.05). *n* = 15.

**Table 2:** Effects of different amounts of *Acremonium terricola* culture on milk performance in lactating dairy cows.

Items	ATC Supplementation, g/d per Head				SEM	*p*-Value			
	0	30	60	300		G	G × T	L	Q
Milk yield, kg/d									
Average	27.33 ^c^	28.98 ^b^	30.15 ^a^	30.35 ^a^	0.376	<0.01	0.98	<0.01	<0.01
0–30 d	28.56 ^b^	29.75 ^ab^	30.87 ^a^	31.00 ^a^	0.918	0.01			
30–60 d	27.53 ^b^	29.28 ^ab^	30.38 ^a^	30.57 ^a^	0.896	<0.01			
60–90 d	25.90 ^b^	27.90 ^a^	29.20 ^a^	29.49 ^a^	0.882	<0.01			
FCM yield, kg/d									
Average	32.73 ^b^	34.95 ^ab^	36.73 ^a^	36.95 ^a^	0.947	<0.01	1.00	<0.01	0.04
0–30 d	33.93	36.34	37.64	37.99	1.552	0.05			
30–60 d	33.30	34.98	37.22	36.95	1.451	0.06			
60–90 d	30.95 ^b^	33.52 ^ab^	35.32 ^a^	35.92 ^a^	1.350	0.02			
ECM, kg/d									
Average	31.36 ^c^	33.41 ^b^	34.88 ^a^	35.18 ^a^	0.623	<0.01	0.98	<0.01	<0.01
0–30 d	33.26	34.89	35.96	36.13	1.107	0.05			
30–60 d	31.38 ^b^	33.54 ^ab^	35.46 ^a^	35.30 ^a^	1.035	0.01			
60–90 d	29.44 ^b^	31.80 ^ab^	33.24 ^a^	34.10 ^a^	0.963	<0.01			
Milk yield/DMI									
Average	1.26 ^c^	1.32 ^b^	1.38 ^ab^	1.39 ^a^	0.018	<0.01	0.99	<0.01	0.09
0–30 d	1.31 ^b^	1.35 ^ab^	1.40 ^a^	1.42 ^a^	0.021	0.01			
30–60 d	1.29 ^b^	1.36 ^ab^	1.41 ^a^	1.41 ^a^	0.020	0.01			
60–90 d	1.19 ^b^	1.26 ^ab^	1.33 ^a^	1.34 ^a^	0.017	<0.01			
FCM/DMI									
Average	1.51 ^b^	1.60 ^ab^	1.68 ^a^	1.69 ^a^	0.037	0.01	1.00	0.02	0.17
0–30 d	1.55	1.65	1.70	1.74	0.073	0.06			
30–60 d	1.56	1.63	1.73	1.70	0.068	0.08			
60–90 d	1.42	1.51	1.61	1.63	0.063	0.06			
ECM/DMI									
Average	1.45 ^b^	1.53 ^b^	1.60 ^a^	1.61 ^a^	0.032	<0.01	0.98	<0.01	0.07
0–30 d	1.52	1.59	1.63	1.66	0.053	0.05			
30–60 d	1.52 ^b^	1.56 ^ab^	1.65 ^a^	1.66 ^a^	0.050	0.01			
60–90 d	1.35 ^b^	1.43 ^ab^	1.52 ^a^	1.55 ^a^	0.046	0.01			

ATC, *Acremonium terricola* culture; SEM, standard error; ECM, energy-corrected milk; FCM, fat-corrected milk; DMI, dry-matter intake; Milk yield/DMI, kg of milk yield/kg of DMI; FCM/DMI, kg of FCM/kg of DMI; ECM/DMI, kg of ECM/kg of DMI; ECM = 0.3246 × milk yield + 13.86 × milk fat yield + 7.04 × milk protein yield; FCM = milk yield × 0.432 + milk fat yield × 16. The effects included group effect (G) and the interaction effect of group and time (G × T), as well as linear (L) and quadratic effects (Q). Different lowercase letters in the same row show significant differences (*p* < 0.05). *n* = 15.

**Table 3:** Effects of different amounts of *Acremonium terricola* culture on milk composition in lactating dairy cows.

Items	ATC Supplementation, g/d per Head				SEM	*p*-Value			
	0	30	60	300		G	G × T	L	Q
Milk protein, %									
Average	3.81	3.83	3.82	3.83	0.064	0.99	0.80	0.48	0.37
0–30 d	3.98	3.93	3.88	3.86	0.105	0.37			
30–60 d	3.71	3.81	3.88	3.83	0.098	0.22			
60–90 d	3.73	3.74	3.70	3.80	0.091	0.46			
Milk protein yield, kg/d									
Average	1.04 ^b^	1.11 ^a^	1.15 ^a^	1.16 ^a^	0.026	<0.01	0.85	<0.01	<0.01
0–30 d	1.14	1.17	1.20	1.20	0.042	0.30			
30–60 d	1.02 ^b^	1.12 ^ab^	1.18 ^a^	1.17 ^a^	0.039	0.01			
60–90 d	0.97 ^b^	1.04 ^ab^	1.08 ^a^	1.13 ^a^	0.037	0.01			
Milk fat, %									
Average	4.73	4.77	4.83	4.84	0.152	0.94	1.00	0.88	0.99
0–30 d	4.68	4.87	4.85	4.91	0.270	0.51			
30–60 d	4.79	4.70	4.86	4.77	0.253	0.65			
60–90 d	4.71	4.74	4.79	4.85	0.235	0.69			
Milk fat yield, kg/d									
Average	1.29 ^b^	1.38 ^ab^	1.46 ^a^	1.47 ^a^	0.053	0.04	1.00	0.03	0.15
0–30 d	1.33	1.45	1.50	1.52	0.087	0.11			
30–60 d	1.32	1.38	1.48	1.46	0.081	0.16			
60–90 d	1.22	1.32	1.40	1.43	0.076	0.07			
Milk lactose, %									
Average	5.08	5.09	5.04	5.06	0.034	0.73	0.95	0.18	0.45
0–30 d	5.15	5.12	5.08	5.10	0.060	0.41			
30–60 d	4.99	5.05	5.02	5.03	0.056	0.48			
60–90 d	5.09	5.09	5.01	5.04	0.052	0.29			
Milk lactose yield, kg/d									
Average	1.39 ^c^	1.48 ^b^	1.52 ^ab^	1.54 ^a^	0.02	<0.01	<0.01	<0.01	0.04
0–30 d	1.47 ^b^	1.52 ^ab^	1.57 ^a^	1.58 ^a^	0.03	0.02			
30–60 d	1.38 ^b^	1.48 ^a^	1.52 ^a^	1.54 ^a^	0.03	<0.01			
60–90 d	1.32 ^b^	1.42 ^a^	1.46 ^a^	1.49 ^a^	0.03	<0.01			
Milk urea nitrogen, mg/dL									
Average	17.23	16.70	17.13	18.18	0.715	0.49	0.95	0.84	0.74
0–30 d	14.76	14.26	15.33	17.07	1.105	0.10			
30–60 d	16.32	15.29	15.70	16.10	1.270	0.56			
60–90 d	20.61	20.56	20.37	21.38	1.105	0.54			
SCC, ×1000/mL									
Average	68.06 ^a^	52.23 ^b^	47.90 ^bc^	43.68 ^c^	2.705	<0.01	0.42	<0.01	<0.01
0–30 d	67.23 ^a^	58.60 ^ab^	52.98 ^bc^	41.85 ^c^	4.809	<0.01			
30–60 d	70.73 ^a^	44.73 ^b^	44.64 ^b^	45.25 ^b^	4.496	<0.01			
60–90 d	66.23 ^a^	53.35 ^ab^	46.09 ^b^	43.96 ^b^	4.182	<0.01			

ATC, *Acremonium terricola* culture; SEM, standard error; SCC, somatic cell count. The effects included group effect (G) and the interaction effect of group and time (G × T), as well as linear (L) and quadratic effects (Q). Different lowercase letters in the same row show significant differences (*p* < 0.05). *n* = 15.

## Data Availability

The data presented in this study are available in this manuscript. The raw reads were deposited in the NCBI Sequence Read Archive (SRA) database (Accession Numbers: SRR16916794–SRR16916841).

## Supplementary Materials

The following are available online at https://www.mdpi.com/article/10.3390/antiox11010175/s1, Table S1: Functional composition of *Acremonium terricola* culture; Table S2: Ingredients and nutrient composition of the experiment diet; Table S3: Sample information and sequencing statistics.
