# Supplementary material for: Acremonium terricola Culture’s Dose–Response Effects on Lactational Performance, Antioxidant Capacity, and Ruminal Characteristics in Holstein Dairy Cows
Source: Antioxidants (Basel). 2022 Jan 17;11(1):175. doi: 10.3390/antiox11010175 (PMC8772898; doi:10.3390/antiox11010175)
Supplement: Supplementary file 1 [file antioxidants-11-00175-s001.zip › Supplementary Table S1.pdf]

**Supplementary Table S1** Functional composition of *Acremonium terricola* cultures, dry matter basis

| Items | Value, % |
|---|---|
| Dry matter (%, air dry basis) | 94.40 |
| Ether extract | 3.06 |
| Crude protein | 24.53 |
| D-mannitol | 84.50 |
| Galactomannan | 44.60 |
| 3′-deoxyadenosine | 0.43 |
| Ergosterol | 0.60 |
| Total amino acids | 17.27 |
| Aspartic acid | 1.79 |
| Threonine | 0.72 |
| Serine | 0.82 |
| Glutamic acid | 3.30 |
| Proline | 1.50 |
| Glycine | 0.76 |
| Alanine | 0.97 |
| Cystine | 0.29 |
| Valine | 0.90 |
| Methionine | 0.26 |
| Isoleucine | 0.74 |
| Leucine | 1.49 |
| Tyrosine | 0.36 |
| Phenylalanine | 0.88 |
| Lysine | 0.92 |
| Histidine | 0.46 |
| Arginine | 1.11 |
