# Supplementary material for: Acremonium terricola Culture’s Dose–Response Effects on Lactational Performance, Antioxidant Capacity, and Ruminal Characteristics in Holstein Dairy Cows
Source: Antioxidants (Basel). 2022 Jan 17;11(1):175. doi: 10.3390/antiox11010175 (PMC8772898; doi:10.3390/antiox11010175)
Supplement: Supplementary file 1 [file antioxidants-11-00175-s001.zip › Supplementary Table S2.pdf]

Supplementary Table S2 Ingredients and nutrient composition of the experiment diet

Items	Value
Ingredient, % of dry matter	
Alfalfa	9.34
Whole corn silage	25.21
Alfalfa silage	2.12
Flaked corn	17.82
Corn	12.73
Soybean meal	15.28
Soybean hull	4.24
corn gluten meal	2.55
Cottonseed	6.37
Fat power	1.27
$Ca(HCO_3)_2$	0.64
Molasses	1.05
5% Premix	1.38
Chemical composition [1], % of dry matter	
Dry matter, % as fed	48.90
Ether extract	5.03
Crude protein	15.18
Neutral detergent fibre	31.59
Acid detergent fibre	17.63

[1] All contents were measured value.
