# Supplementary material for: Acremonium terricola Culture’s Dose–Response Effects on Lactational Performance, Antioxidant Capacity, and Ruminal Characteristics in Holstein Dairy Cows
Source: Antioxidants (Basel). 2022 Jan 17;11(1):175. doi: 10.3390/antiox11010175 (PMC8772898; doi:10.3390/antiox11010175)
Supplement: Supplementary file 1 [file antioxidants-11-00175-s001.zip › Supplementary Table S3.pdf]

Supplementary Table S3. Sample information and sequencing statistics

Groups	Days, d	Number of sequences	Good's coverage, %
0 g/d	0	53408 ± 7466	99.05 ± 0.07
	90	56622 ± 6309	99.22 ± 0.08
30 g/d	0	56006 ± 5785	99.07 ± 0.11
	90	53420 ± 3163	99.12 ± 0.06
60 g/d	0	57964 ± 4083	99.11 ± 0.08
	90	52988 ± 2973	99.19 ± 0.10
300 g/d	0	57537 ± 4028	99.15 ± 0.04
	90	56076 ± 4152	99.25 ± 0.06
